# Molnupiravir and Nirmatrelvir/Ritonavir: Tolerability, Safety, and Adherence in a Retrospective Cohort Study

**DOI:** 10.3390/v15020384

**Published:** 2023-01-28

**Authors:** Maria Mazzitelli, Daniele Mengato, Lolita Sasset, Anna Ferrari, Samuele Gardin, Vincenzo Scaglione, Nicola Bonadiman, Lucrezia Calandrino, Silvia Cavinato, Sabrina Trivellato, Francesca Venturini, Anna Maria Cattelan

**Affiliations:** 1 Infectious and Tropical Diseases Unit, Padua University Hospital, 35128 Padua, Italy; 2 Hospital Pharmacy Department, Padua University Hospital, 35128 Padua, Italy

**Keywords:** molnupiravir, nirmatrelvir/ritonavir, tolerability, adverse drug reaction, adherence, COVID-19

## Abstract

Background. Molnupiravir (MOL) and nirmatrelvir/ritonavir (NIR) were recently approved for the early treatment of COVID-19, but real-life data on tolerability, safety, and adverse events (AEs) are still scarce. Methods. We conducted a retrospective cohort study including all patients who were prescribed MOL and NIR at the Infectious Diseases Unit of Padua University Hospital, between January and May 2022. Demographic, clinical, and safety variables were recorded. Results. We included 909 patients, 48.3% males and 95.2% vaccinated against SARS-CoV-2. The median age was 73 (IQR: 62–82) years. MOL and NIR were prescribed in 407 (44.8%) and 502 (55.2%) patients, respectively. Overall, 124/909 (13.6%) patients experienced any AEs following antivirals intake: 98/124 (79%) patients reporting adverse events presented grade 1 AEs, 23/124 (18.5%) grade 2 AEs and 3 (2.5%) grade 3 AEs. Treatment discontinuation was recorded in 4.8% of patients. AEs were significantly higher in women, in patients treated with NIR compared to MOL and in people who were not vaccinated. Conclusions. In our real-life setting, AEs were higher than those reported by clinical trials, and were particularly associated with NIR use and with not being vaccinated. Further analyses are needed to better assess safety of oral antivirals and to define which patient’s profile may benefit most from MOL and NIR.

## 1. Introduction

Since December 2020, huge efforts have been made to counteract spreading of Severe Acute Respiratory Syndrome Coronavirus 2 (SARS-CoV-2) pandemic, especially in terms of implementation of new prevention and treatment strategies. Most patients with SARS-CoV-2 infection remains asymptomatic, but some may develop severe forms of Coronavirus Diseases 19 (COVID-19), requiring hospitalization and intensive care admission [1]. Vaccination provided extensive benefits in the prevention of severe COVID-19; however, some categories of patients may remain at risk of hospitalizations and of developing severe COVID-19 and related complications [2,3] Those subjects are more likely to have an advanced age and multiple comorbidities, such as immunosuppression, cardiovascular diseases, pulmonary diseases, neurological disorders, diabetes, renal disease, and obesity [3]. 

Recently and, two antiviral oral drugs, molnupiravir and nirmatrelvir/ritonavir, also active against the more recent Omicron SARS-CoV-2 variants, have received the approval with an emergency procedure and made available for the outpatient setting for the early treatment of COVID-19, in adult, non-hospitalized patients at high risk for disease progression [4]. 

Molnupiravir is a high genetic barrier drug that is incorporated into the viral SARS-CoV-2 RNA by the viral RNA polymerase and block viral replication by causing errors due to incorporation of different bases [5]. Molnupiravir efficacy was granted and supported by a phase 3 clinical trial (MOVe-OUT), including 775 patients (not-vaccinated and not requiring oxygen supplementation) who were randomized to receive molnupiravir or placebo for 5 days, within 5 days from SARS-CoV-2 symptom’s onset [5]. Results from this study confirmed that molnupiravir, compared to the placebo, reduced both the risk of being hospitalized and of death for COVID-19 [5]. 

Nirmatrelvir/ritonavir is an oral antiviral agent targeting the SARS-CoV-2 3-chymotrypsin-like cysteine protease enzyme, which is crucial for the viral replication cycle [6]. Nilmatrelvir was boosted with ritonavir, to improve its bioavailability and pharmacokinetics [6]. Efficacy of such combination was demonstrated in the EPIC-HR clinical trials, in which 2246 participants (not vaccinated and with risk factors for COVID-19 progression) were randomized to receive nirmatrelvir/ritonavir or placebo [6]. Subjects who received nirmatrelvir/ritonavir exhibited a lower risk of being hospitalized and of developing the severe form of the diseases [6]. Since their release (January 2022), they have proven to be an effective strategy to prevent hospitalizations even in the real-life setting [7,8]. Although clinical trials granted their efficacy and safety [5,6], real life data on the possible adverse events (AEs), tolerability and safety of these drugs are scarce and available only in small cohort of patients [9,10,11,12]. 

Therefore, aim of this study was to evaluate in a real life setting the overall tolerability and safety of molnupiravir and nirmatrelvir/ritonavir by retrospectively assessing the proportions and type of adverse events detected in clinical practice. Secondly, we assessed and described both the proportion and type of side effects and the proportion of patients who did not complete treatment, also investigating factors (demographic and clinical) associated with antiviral agent AEs. 

## 2. Materials and Methods

We conducted a retrospective study on all patients who were consecutively referred to the outpatient clinic for the early treatment of COVID-19 at the Infectious Diseases Unit of the University Hospital of Padua.

We included all adult patients who were prescribed molnupiravir or nirmatrelvir/ritonavir from 30 January 2022 (date in which the drugs were available in our center) to 30 May 2022. According to the Italian Drug Agency indications, molnupiravir and nirmatrelvir/ritonavir were prescribed to all patients with recent symptom onset (≤5 days), no need of oxygen support, and having a high risk of COVID-19 progression (i.e., the presence of at least one of the following conditions: diabetes with organ damage or uncontrolled, body mass index > 30 kg/m^2^, chronic kidney disease, lung disease, cardiovascular disease, cancer, immunodeficiency status). According to the same criteria, patients who had an estimated glomerular filtration rate lower than 30 mL/min/1.73 m^2^, pregnant women and patients with end stage liver disease were excluded. 

As a part or clinical practice, we asked patients who came back for SARS-CoV-2 testing at the end of treatment to give us back the antiviral pills they did not take for whatever reason. Reasons and rate for discontinuation were recorded. 

Patients’ demographics (age, gender), clinical data (comorbidities and comedications, SARS-CoV-2 related symptoms, date of symptom onset, time in between symptom onset and SARS-CoV-2 diagnosis, time in between SARS-CoV-2 diagnosis and drug prescription), and type and number of AEs which have been developed within 7 days of follow-up were collected. Patients, according to AIFA requirements/dispositions, were followed up for 30 days. To avoid possible COVID-19 related confounding factors, all signs, and symptoms of new onset since the antivirals administration were considered AEs. Each AE detected was also recorded in the National Pharmacovigilance Network and graded by severity as follows: grade 1 = mild, grade 2 = moderate, grade 3 = severe, grade 4 = life-threatening, grade 5 = death related to adverse event. Information about clinical outcome, treatment interruption, possible access to emergency department (ED), and hospitalizations were retrieved from medical health records. Clinical cure was defined as resolution of symptoms and alterations due to COVID-19. Data were anonymized and subsequently analyzed through R software. Qualitative variables were expressed as absolute numbers and percentages and compared using the Chi-square test. Quantitative variables were expressed as mean or median and compared by T-test or Mann-Whitney test whenever it was more appropriate. Level of significance was set up at <0.05. Logistic regression was implemented to assess factors associated with the presence of ADRs. The odds ratio (OR) and 95% confidence interval (CI) were calculated as effect measure for risk association. The protocol received institutional board approval (n°0002323, 14 January 2022). Moreover, in order to reduce possible bias induced by different criteria that clinicians could have applied to prescribe either molnupiravir or nirmatrelvir/ritonavir, we perform a logistic regression for each drug, in order to explore factors potentially AEs associated with each specific antiviral agent. 

## 3. Results

**Figure 1:**
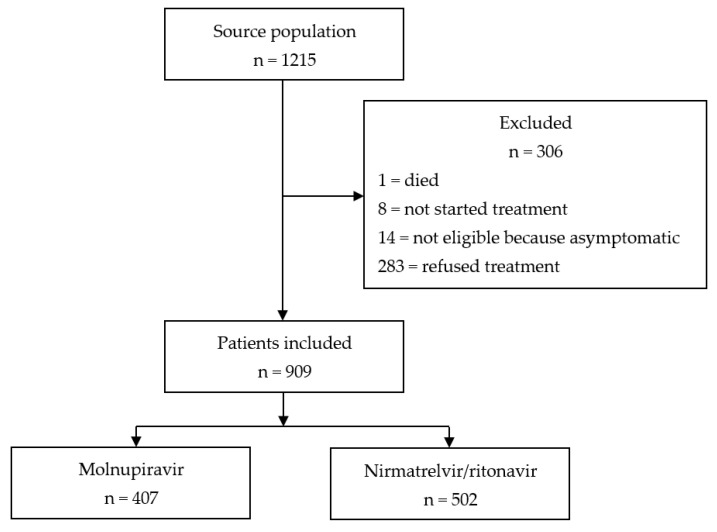
Patient’s flow.

From 21 January to 30 June 2022, 918 consecutive patients were prescribed oral antiviral agents in our COVID-19 outpatient’s clinic. One patient died before initiating treatment, 14 were excluded because of lack of symptoms, 8 patients received the drug but did not start antiviral therapy because of symptom relieve, and 283 patients refused treatment (patient’s flow, Figure 1). Therefore, 909 patients were included in the present analysis. Molnupiravir and nirmatrelvir/ritonavir were prescribed to 407/909 (44.8%) and 502/909 (55.2%) patients, respectively. The full characteristics (demographics and clinic) overall and by treatment group are shown in Table 1. The median age was of 73 (interquartile range, IQR: 62–82) years, 439/909 (48.3%) participants were male, 865/909 (95.2%) received vaccination against SARS-CoV-2. Patients who were not vaccinated experienced a mean number of symptoms significantly higher than those who were vaccinated (2.8, SD: 1.4 vs. 3.5, SD 1.4, *p* = 0.011). All patients started treatment within a median of 1 day (IQR: 0–2) from symptoms onset. Similarly, median time from SARS-CoV-2 diagnosis and treatment was of 1 day (IQR: 0–2). The median number of SARS-CoV-2 symptoms at presentation was significantly higher (*p* = 0.02) in the group that received nirmatrelvir/ritonavir, compared to that receiving molnupiravir.

Patients treated with molnupiravir were significantly older (*p* < 0.05) and with a larger number of comorbidities (*p* = 0.04) than patients treated with nirmatrelvir/ritonavir. Indeed, prevalence of obesity was significantly higher in the group that received nirmatrelvir/ritonavir, while cardiovascular diseases, renal diseases, and neurological disorders were significantly more represented in the group that received molnupiravir. 

The prevalence of patients who were on polypharmacy (i.e., intake of 5 or more chronic comedications in the same patients), excluding vitamin D, in the group receiving molnupiravir we had 200/407 (49.1%) on polypharmacy, a significantly higher proportion compared to that receiving nirmatrelvir/ritonavir (75/502, 14.9%), *p* < 0.05. Moreover, median numbers of comedication were higher in the group receiving molnupiravir, 4 (IQR: 3–6), than in that receiving nirmatrelvir/ritonavir, 2 (IQR 2–4). In the group receiving molnupiravir, 46/407 (11.3%) patients were receiving 55 comedications causing red flag drug interactions if administer with ritonavir. These drugs were: amiodaron in 7 cases, phenobarbital, or carbamazepine in 8 cases, and clopidogrel 40 cases. 

Type, prevalence, and outcomes of the different AEs are reported in Table 2 and Supplementary Table S1. Overall, 124/909 (13.6%) patients experienced any AEs following antivirals intake: 98/124 (79%) patients reporting adverse events presented grade 1 AEs, 23/124 (18.5%) grade 2 AEs and 3 (2.5%) grade 3 AEs. Regarding grading of side effects, we did not detect any differences between molnupiravir and nirmatrelvir/ritonavir for AEs of grade 2 and 3, but for grade 1 AEs were more frequent in patients who were prescribed nirmatrelvir/ritonavir, compared to those receiving molnupiravir (80/502, 13.9% vs. 18/407, 4.4%, *p* < 0.05). 

The most reported side effects were: dysgeusia (7.4%), bloating (2.3%), diarrhea (2.1%), and nausea/vomiting (2%). Three patients (0.3%) reported a severe hypersensitivity reaction (2 treated with molnupiravir and 1 treated with nirmatrelvir/ritonavir). In patients reporting adverse events, we did not detect any drug interactions between chronic comedication intake and the antiviral agents we described.

The proportion of patients who experienced side effects was significantly higher in the nirmatrelvir/ritonavir group, compared to the molnupiravir one (96/502, 19.1% vs. 28/407, 6.9%, *p* < 0.05). Prevalence of dysgeusia and diarrhea was also significantly higher in the group receiving nirmatrelvir/ritonavir that that reported in the molnupiravir group. 

Overall, twenty-seven (3%) patients reported access to ED, 4 (0.3%) were hospitalised, and 2 (0.2%) patients died. In terms of such treatment outcomes, no differences were observed between the two antivirals. Among 21 people who had access to ED and were not hospitalised, we had 3 patients who reported allergy to the prescribed oral antiviral agents, and 18 for abdominal pain and/or diarrhoea. For these reasons, even if doctors recognised these as a grade 2 adverse events, they made the decision to discontinue the medications. 

A further analysis of clinical records documented that hospitalizations and deaths were not related to antivirals, but to the worsening of COVID-19 (onset of dyspnoea and pneumonia requiring oxygen support in 5 cases, and a concomitant pulmonary thromboembolism in 1 case). 

Clinical cure within 5 days from the antiviral intake was reported in 68% patients, with a higher proportion of subject in the molnupiravir group, compared to the nirmatrelvir/ritonavir one (293, 71.9% vs. 325, 64.8%, *p* = 0.02).

According to SARS-CoV-2 vaccination status, AEs were observed in 112/865 (12.9%) patients who were vaccinated and in 12/44 (27.3%) patients who were not vaccinated (*p* = 0.007). Due to side effects, forty-four patients (4.8%) did not complete the prescribed treatment and gave us back pills not taken: 26 (2.8%) patients took ≤50% of the expected antiviral doses, while 18 (2%) patients took from 60 to 90% of the expected doses. 

Univariate analysis showed that the following factors were significantly associated with AEs: age (*p* < 0.05), gender (*p* < 0.05), being vaccinated for SARS-CoV-2 (*p* < 0.05), the type of antiviral drug (*p* < 0.05), and the number of symptoms at presentation (*p* = 0.045). Multivariable analysis confirmed statistical significance for gender (OR: 0.49, CI: 0.32–0.75, *p* = 0.001), the type of antiviral drug (OR: 0.39, CI: 0.23–0.64, *p* < 0.001), and being vaccinated for SARS-CoV-2 (OR: 0.43, CI 0.2–0.9, *p* = 0.02). According to antiviral drug, people treated with nirmatrelvir/ritonavir showed a probability of 61% higher than those treated with molnupiravir of experiencing AEs. 

When univariate analysis was performed to explore factors associated with AEs in patients who received molnupiravir (Table 3), we detected that gender (*p* < 0.05), age (*p* < 0.05), being vaccinated for SARS-CoV-2 (*p* < 0.05), and time from symptom onset (*p* < 0.05) were factors significantly associated with the presence of AEs. When multivariable was performed, the only associations retained were those between AEs and gender (OR: 0.24, CI 0.08–0.69, *p* = 0.008), and between AEs and being vaccinated against SARS-CoV-2 (OR: 0.14, CI 0.03–0.7, *p* = 0.01).

Similarly, when univariate analysis was performed to explore factors associated with AEs in patients who received nirmatrelvir/ritonavir (Table 4), we detected that gender (*p* < 0.05), age (*p* < 0.05), and shortness of breath as symptom of presentation (*p* < 0.05) were significantly associated with the presence of AEs. When multivariable was performed, the only association which was retained was that between AEs and gender (OR: 0.54, CI 0.32–0.90, *p* = 0.02).

## 4. Discussion

To the best of our knowledge, this study is among the first and largest studies assessing in a real-life setting, the tolerability and safety of the new antiviral agents approved for the early treatment of COVID-19 (molnupiravir and nirmatrelvir/ritonavir) [9,10,11]. Moreover, this is the first study assessing antiviral adherence, by assessing the pill burden. 

Evidence emerging from our study, albeit limited, suggested an overall higher frequency of adverse events than that found in clinical trials, especially in patients treated with nirmatrelvir/ritonavir [5,6]. Different prevalence of AEs may be attributed to differences between the real-life population and that included in clinical trials, especially regarding age. Indeed, in clinical trials, median age of subjects recruited, and number of comorbidities were lower than that observed in our cohort in which 74.6% of patients were older than 65 years and with a high prevalence of comorbidities (especially cardiovascular, pulmonary, and metabolic). 

Our cohort, in contrast to the cohorts of registration studies, presented a very high percentage of patients who received SARS-CoV-2 vaccination [5,6]. Despite a limited number of people who were not vaccinated in our cohort, we observed a protective association of being vaccinated and experiencing AEs due to antivirals. We can hypothesize that people who were not vaccinated having a higher burden of symptoms were also more likely to feel AEs. This hypothesis is indeed supported by our results.

Regarding nirmatrelvir/ritonavir, we observed a high frequency of cases of dysgeusia, much more frequent than reported in the literature [6]. Specifically, all patients of our cohort disclosed a “metallic taste”, following intake of nirmatrelvir/ritonavir. Higher proportion of side effects in the nirmatrelvir/ritonavir group is likely related to ritonavir [13]. Of note, in our analysis molnupiravir was associated with fewer side effects than nirmatrelvir/ritonavir, even if administered to an older and a “more symptomatic” population. These findings may suggest that molnupiravir may be considered by clinicians a safer early treatment choice than nirmatrelvir/ritonavir in elderly people affected by SARS-CoV-2 infection who often are on polypharmacy and therefore exposed to the risk of drug-drug interactions enhanced by ritonavir. Moreover, in our cohort of patients, molnupiravir demonstrated a favorable efficacy with higher rate of clinical cure than nirmatrelvir/ritonavir. It may be suggested that in this evolving scenario with less pathogen viral variants and high proportion of vaccinated people, the use of molnupiravir seems to be favored also by a lower rate of AEs [14]. 

Of note, our data recorded a treatment interruption in almost 5% patients, a far higher percentage when considering similar Italian studies (0.5% and 0.2%, respectively) [9,10,11]. This difference could be explained by ages of the different population. Our cohort indeed was older and with a greater burden of comorbidities. Moreover, it could be also related to the strict adherence monitoring (pill counts) adopted in our clinical practice.

In our cohort, seemed that also female gender was keen to develop more frequently adverse events. This data is in line with several studies, also conducted for other drugs in real-life settings, demonstrating how females are more likely to experience drug side effects [15,16]. Moreover, it must be considered that clinical trials tend to recruit young male, limiting access to women and women with motherhood wish.

This study is somewhat limited by its retrospective nature and by the lack of a control group of patients who did not receive any treatment. Moreover, we are not able to assess any biochemical toxicity since we did not perform blood tests at the baseline and during follow-up for all our patients. Further analysis on larger cohorts, also considering possible confounding factors, need to be performed in order both to better assess the real-life safety profile of these new antivirals and to define which patients may benefit most from each of them. Such studies will help clinicians to better personalize, and tailor COVID-19 treatment based on patient’s profile.

## Figures and Tables

**Table 1:** Baseline characteristics of the study population.

Characteristic		Overall N = 909	Molnupiravir N = 407	Nirmatrelvir/Ritonavir N = 502	*p* Value
Age, median (IQR)		73 (62–82)	80 (71–86)	68 (56–76)	<0.05
Gender, male, n (%)		439 (48.3)	198 (48.6)	241 (48.0)	0.85
Vaccination against SARS-CoV-2, n (%)		865 (95.2)	391 (96.0)	474 (94.4)	0.25
Age ≥ 65 years, n (%)		674 (74.2)	353 (86.7)	321 (63.9)	<0.05
Obesity, n (%)		125 (13.7)	42 (10.3)	83 (16.5)	<0.05
Diabetes, n (%)		175 (19.3)	84 (20.6)	91 (18.1)	0.33
Cardiovascular diseases, n (%)		467 (51.4)	271 (66.6)	196 (39.0)	<0.05
Cancer, n (%)		60 (6.6)	25 (6.1)	35 (7.0)	0.62
Renal disease, n (%)		33 (3.6)	22 (5.4)	11 (2.2)	<0.05
Lung disease, n (%)		162 (17.8)	65 (16.0)	97 (19.3)	0.20
Neurological disease, n (%)		59 (6.5)	38 (9.3)	21 (4.2)	<0.05
Immunosuppression, n (%)		75 (8.3)	29 (7.1)	46 (9.2)	0.27
Other comorbidities, n (%)		96 (10.6)	53 (13.0)	43 (8.6)	0.03
N comorbidities/patients, median (IQR)		1 (1–2)	1 (1–2)	1 (1–2)	<0.05
N comorbidities/patients, mean (±SD)		1.4 (1.0)	1.6 (1.0)	1.2 (0.9)	0.04
Time from symptom onset to diagnosis, days, median (IQR)		1 (0–2)	1 (0–2)	1 (0–2)	0.21
Time from symptom onset to treatment, days, median (IQR)		3 (2–4)	3 (2–4)	3 (2–4)	0.24
Number of symptoms at presentation, median (IQR)		3 (2–4)	3 (2–3)	3 (2–4)	0.02
Symptoms at presentation, n (%)	Fever	509 (56.0)	219 (53.8)	290 (57.8)	0.23
	Shortness of breath	9 (1.0)	5 (1.2)	4 (0.8)	0.51
	Headache	183 (20.1)	119 (23.7)	64 (15.7)	<0.05
	Anosmia	16 (1.8)	8 (2.0)	8 (1.6)	0.67
	Ageusia	21 (2.3)	6 (1.5)	15 (3.0)	0.13
	Sore throat	319 (35.1)	135 (33.2)	184 (36.7)	0.27
	Cold	143 (15.7)	54 (13.3)	89 (17.7)	0.07
	Myalgia	259 (28.5)	95 (23.3)	164 (32.7)	<0.05
	Tachypnoea	9 (1.0)	5 (1.2)	4 (0.8)	0.51
	Asthenia	392 (43.1)	186 (45.7)	206 (41.0)	0.16
	Gastrointestinal disorders	62 (6.8)	31 (7.6)	31 (6.2)	0.39

N = number, IQR = interquartile range, SD = Standard deviation.

**Table 2:** Type, prevalence, and outcomes of adverse events.

Characteristic		Overall N = 909	Molnupiravir N = 407	Nirmatrelvir/Ritonavir N = 502	*p* Value
Side effects, yes, n (%)		124 (13.6)	28 (6.9)	96 (19.1)	<0.05
Side effect/patient, median (IQR)		1 (1–1)	1 (1–1)	1 (1–1)	0.98
Patients with ADE >1, n (%)		24 (2.6)	6 (1.5)	18 (3.6)	0.06
Type of side effects, n (%)	Dysgeusia	67 (7.4)	5 (1.2)	62 (12.4)	<0.05
	Bloating	21 (2.3)	7 (1.7)	14 (2.8)	0.29
	Allergy	3 (0.3)	2 (0.5)	1 (0.2)	0.44
	Nausea/vomiting	18 (2.0)	8 (2.0)	10 (2.0)	0.98
	Diarrhoea	19 (2.1)	4 (1.0)	15 (3.0)	<0.05
	Headache	9 (0.9)	2 (0.5)	7 (1.4)	0.17
	Other	11 (1.2)	5 (1.2)	6 (1.2)	0.96
Adverse event grading	Grade 1	98 (10.8)	18 (4.4)	80 (13.9)	<0.05
	Grade 2	23 (2.5)	8 (1.9)	15 (2.9)	0.398
	Grade 3	3 (0.33)	2 (0.4)	1 (0.2)	0.589
Completed treatment, n (%)		865 (95.6)	390 (95.8)	475 (94.6)	0.40
Median proportion of doses taken, median (IQR)		100 (100–100)	100 (100–100)	100 (100–100)	0.40
Proportion of doses taken, mean (±SD)		97.7 (11.2)	98.0 (10.6)	97.6 (11.7)	0.52
Proportion of subjects taken expected antiviral doses:	≤50%, n (%)	26 (2.9)	9 (2.2)	17 (3.4)	0.42
	>50%, n (%)	883 (97.1)	398 (97.8)	485 (96.6)	0.42
Access to ED, yes, n (%)		27 (3.0)	14 (3.4)	13 (2.6)	0.45
Admission, yes, n (%)		4 (0.4)	1 (0.2)	3 (0.6)	0.42
Death, yes, n (%)		2 (0.2)	2 (0.5)	0 (0.0)	0.11
Symptom’s’ resolution within 5 days, n (%)		618 (68.0)	293 (71.9)	325 (64.8)	0.02

N = number, IQR = interquartile range, SD = Standard deviation, ED = emergency department.

**Table 3:** Univariate and multivariable analysis for factors associated with AEs in patients who received molnupiravir.

Characteristic		Univariate Analysis				Multivariable Analysis			
		OR	Lower 95% CI	Upper 95% CI	*p* Value	OR	Lower 95% CI	Upper 95% CI	*p* Value
Age, median (IQR)		0.973	0.946	1.000	0.050	0.993	0.939	1.050	0.803
Gender, male, n (%)		0.328	0.136	0.790	<0.05	0.242	0.084	0.693	0.008
Vaccination against SARS-CoV-2, n (%)		0.138	0.044	0.429	<0.05	0.148	0.031	0.704	0.016
Age ≥ 65 years, n (%)		0.345	0.144	0.829	0.02	0.243	0.042	1.390	0.112
Obesity, n (%)		1.050	0.302	3.620	0.943	0.279	0.03	2.140	0.219
Diabetes, n (%)		0.623	0.210	1.850	0.393	0.581	0.1150	2.930	0.511
Cardiovascular diseases, n (%)		2.43	0.903	6.540	0.072	2.560	0.541	12.11	0.236
Cancer, n (%)		0.548	0.071	4.200	0.563	0.232	0.018	2.890	0.256
Renal disease, n (%)		0.000	0.000	Inf	0.986	0	0	inf	0.993
Lung disease, n (%)		1.160	0.423	3.160	0.778	0.715	0.112	4.570	0.723
Neurological disease, n (%)		1.180	0.339	4.100	0.795	1.210	0.199	7.330	0.837
Immunosuppression, n (%)		1.630	0.461	5.760	0.448	1.450	0.226	9.320	0.695
Other comorbidities, n (%)		0.790	0.230	2.710	0.707	0.351	0.0501	2.470	0.293
N comorbidities/patients, median (IQR)		1.070	0.824	1.390	0.601	1.600	0.494	5.200	0.432
Time from symptom onset to diagnosis, days, median (IQR)		1.400	1.020	1.930	0.036	1.250	0.803	1.930	0.326
Time from symptom onset to treatment, days, median (IQR)		1.210	0.905	1.620	0.198	1.070	0.713	1.590	0.755
Number of symptoms at presentation, median (IQR)		1.070	0.824	1.390	0.601	1.040	0.325	3.310	0.953
Symptoms at presentation, n (%)	Fever	1.35	0.618	2.970	0.449	1.450	0.328	6.400	0.626
	Shortness of breath	0.000	0.000	Inf	0.990	0	0	inf	0.993
	Headache	2.31	0.969	5.490	0.058	2.660	0.486	14.600	0.259
	Anosmia	1.97	0.234	16.600	0.533	13.300	0.647	272	0.093
	Ageusia	0.000	0.000	Inf	0.989	0	0	inf	0.096
	Sore throat	0.794	0.340	1.850	0.593	0.974	0.195	4.860	0.975
	Cold	0.772	0.225	2.650	0.681	0.817	0.240	5.618	0.819
	Myalgia	0.889	0.349	2.260	0.804	0.369	0.068	2	0.248
	Tachypnoea	3.47	0.375	32.200	0.273	5.020	0.2740	92	0.277
	Asthenia	1.03	0.478	2.230	0.936	1.34	0.293	6.090	0.708
	Gastrointestinal disorders	0.431	0.057	3.280	0.416	0.29	0.017	4.770	0.386

N = number, IQR = interquartile range, SD = Standard deviation, OR = Odds ratio, CI = confidence in.

**Table 4:** Univariate and multivariable analysis for factors associated with AEs in patients who received nirmatrelvir/ritonavir.

Characteristic		Univariate Analysis				Multivariable Analysis			
		OR	Lower 95% CI	Upper 95% CI	*p* Value	OR	Lower 95% CI	Upper 95% CI	*p* Value
Age, median (IQR)		0.974	0.961	0.988	<0.05	0.980	0.955	1.010	0.11
Gender, male, n (%)		0.621	0.394	0.979	<0.05	0.542	0.326	0.900	0.017
Vaccination against SARS-CoV-2, n (%)		0.694	0.286	1.680	0.418	0.655	0.246	1.750	0.398
Age ≥ 65 years, n (%)		0.416	0.265	0.653	<0.05	0.680	0.319	1.450	0.318
Obesity, n (%)		1.550	0.892	2.70	0.119	1.130	0.502	2.550	0.767
Diabetes, n (%)		0.591	0.308	1.14	0.115	0.656	0.269	1.600	0.353
Cardiovascular diseases, n (%)		1.14	0.728	1.800	0.558	1.640	0.735	3.640	0.228
Cancer, n (%)		0.689	0.260	1.820	0.453	0.826	0.253	2.700	0.751
Renal disease, n (%)		0.939	0.199	4.420	0.936	1.480	0.243	9.04	0.670
Lung disease, n (%)		1.220	0.705	2.100	0.482	0.923	0.071	11.900	0.951
Neurological disease, n (%)		2.200	0.864	5.620	0.098	1.710	0.524	5.590	0.374
Immunosuppression, n (%)		1.200	0.571	2.500	0.636	0.759	0.274	2.100	0.596
Other comorbidities, n (%)		1.330	0.631	2.800	0.454	1.380	0.512	3.730	0.524
N comorbidities/patients, median (IQR)		1.140	0.900	1.430	0.282	0.963	0.517	1.790	0.904
Time from symptom onset to diagnosis, days, median (IQR)		0.993	0.800	1.230	0.946	0.927	0.715	1.20	0.569
Time from symptom onset to treatment, days, median (IQR)		1.060	0.908	1.240	0.448	1.060	0.875	1.280	0.567
Number of symptoms at presentation, median (IQR)		1.120	0.972	1.290	0.118	1.060	0.606	1.84	0.848
Symptoms at presentation, n (%)	Fever	1.350	0.851	2.13	1.35	1.200	0.561	2.580	0.633
	Shortness of breath	2.700	1.400	5.220	<0.05	2.080	0.778	5.570	0.144
	Headache	1.340	0.808	2.210	0.259	1.170	0.518	2.650	0.705
	Anosmia	2.590	0.607	11.000	0.199	2.340	0.366	14.900	0.369
	Ageusia	1.560	0.486	5.010	1.560	0.859	0.187	3.940	0.844
	Sore throat	0.990	0.623	1.570	0.990	0.912	0.423	1.970	0.813
	Cold	0.998	0.557	1.790	0.998	0.946	0.400	2.240	0.899
	Myalgia	1.460	0.919	2.310	1.460	1.180	0.537	2.600	0.679
	Tachypnoea	1.410	0.145	13.700	1.41	0.956	0.085	10.720	0.871
	Asthenia	0.705	0.443	1.120	0.705	0.541	0.252	1.160	0.116
	Gastrointestinal disorders	0.609	0.208	1.780	0.609	0.405	0.112	1.460	0.168

N = number, IQR = interquartile range, SD = Standard deviation, OR = Odds ratio, CI = confidence interval.

## Data Availability

Data supporting findings of this work are available from corresponding author upon a reasonable request.

## Supplementary Materials

The following supporting information can be downloaded at: https://www.mdpi.com/article/10.3390/v15020384/s1, Table S1: Symptoms and grading of side effects.

## References

[1] Zeng H., Ma Y., Zhou Z., Liu W., Huang P., Jiang M., Liu Q., Chen P., Luo H., Chen Y. (2021). Spectrum and Clinical Characteristics of Symptomatic and Asymptomatic Coronavirus Disease 2019 (COVID-19) With and Without Pneumonia. Front. Med..

[2] Lopez Bernal J., Andrews N., Gower C., Robertson C., Stowe J., Tessier E., Simmons R., Roberts R., O’Doherty M., Brown K. (2021). Effectiveness of the Pfizer-BioNTech and Oxford-AstraZeneca vaccines on COVID-19 related symptoms, hospital admissions, and mortality in older adults in England: Test negative case-control study. BMJ.

[3] Hu B., Guo H., Zhou P., Shi Z.L. (2021). Characteristics of SARS-CoV-2 and COVID-19. Nat. Rev. Microbiol..

[4] Vangeel L., Chiu W., De Jonghe S., Maes P., Sletchen B., Raymenants J., Andre E., Leyssen P., Neyts J., Jochmans D. (2022). Remdesivir, Molnupiravir and Nirmatrelvir remain active against SARS-CoV-2 Omicron and other variants of concern. Antivir. Res..

[5] Jayk Bernal A., Gomes da Silva M.M., Musungaie D.B., Kovalchuk E., Gonzalez A., De los Reyes V., Martin-Quiros A., Caraco Y., Williams-Diaz A., Brown M.L. (2022). Molnupiravir for Oral Treatment of COVID-19 in Nonhospitalized Patients. N. Engl. J. Med..

[6] Hammond J., Leister-Tebbe H., Gardner A., Abreu P., Bao W., Wisemandle W., Baniecki M., Hendrick V.M., Damle B., Simón-Campos A. (2022). Oral Nirmatrelvir for High-Risk, Nonhospitalized Adults with COVID-19. N. Engl. J. Med..

[7] Burdet C., Ader F. (2022). Real-world effectiveness of oral antivirals for COVID-19. Lancet.

[8] Scaglione V., Rotundo S., Marascio N., De Marco C., Lionello R., Veneziano C., Berardelli L., Quirino A., Olivadese V., Serapide F. (2022). Lessons learned and implications of early therapies for coronavirus disease in a territorial service centre in the Calabria region: A retrospective study. BMC Infect Dis..

[9] Pontolillo M., Ucciferri C., Borrelli P., Di Nicola M., Vecchiet J., Falasca K. (2022). Molnupiravir as an Early Treatment for COVID-19: A Real Life Study. Pathogens..

[10] Gentile I., Scotto R., Shiano Moriello N., Pinchera B., Villari R., Trucillo E., Ametrano L., Fusco L., Castaldo G., Buonomo A.R. (2022). Nirmatrelvir/Ritonavir and Molnupiravir in the Treatment of Mild/Moderate COVID-19: Results of a Real-Life Study. Vaccines.

[11] De Vito A., Colpani A., Bitti A., Zauli B., Meloni M.C., Fois M., Denti L., Bacciu S., Marcia C., Maida I. (2022). Safety and efficacy of molnupiravir in SARS-CoV-2-infected patients: A real-life experience. J. Med. Virol..

[12] Wen W., Chen C., Tang J., Wang C., Zhou M., Cheng Y., Zhou X., Wu Q., Zhang X., Feng Z. (2022). Efficacy and safety of three new oral antiviral treatment (molnupiravir, fluvoxamine and Paxlovid) for COVID-19a meta-analysis. Ann. Med..

[13] Izcovich A., Siemieniuk R.A., Bartoszko J.J., Ge L., Zeraatkar D., Kum E., Qasim A., Khamis A.M., Rochwerg B., Agoritsas T. (2022). Adverse effects of remdesivir, hydroxychloroquine and lopinavir/ritonavir when used for COVID-19: Systematic review and meta-analysis of randomised trials. BMJ Open.

[14] Najjar-Debbiny R., Gronich N., Weber G., Khoury J., Amar M., Stein N., Goldstein L.H., Saliba W. (2022). Effectiveness of molnupiravir in high-risk patients: A propensity score matched analysis. Clin. Infect. Dis..

[15] Anderson G.D. (2008). Gender differences in pharmacological response. Int. Rev. Neurobiol..

[16] Miller A.M. (2001). Gender-based differences in the toxicity of pharmaceuticals. The food and drug administration’s perspectives. Int. J. Toxicol..

