# Supplementary material for: Molnupiravir and Nirmatrelvir/Ritonavir: Tolerability, Safety, and Adherence in a Retrospective Cohort Study
Source: Viruses. 2023 Jan 28;15(2):384. doi: 10.3390/v15020384 (PMC9962206; doi:10.3390/v15020384)
Supplement: Supplementary file 1 [file viruses-15-00384-s001.zip › viruses-2160244-supplementary.pdf]

Supplementary Materials

Table S1. Symptoms and grading of side effect.

Grade	Dysgeusia N= 67	Bloating N= 21	Allergy N= 3	Nausea/vomiting N=18	Diarrhoea N=19	Headache N=9	Other N=11
1	67	21	0	0	0	0	0
2	0	0	0	18	19	9	11
3	0	0	3	0	0	0	0
4*	0	0	0	0	0	0	0
5*	0	0	0	0	0	0	0

Footnote* No patients reported grade 4 or 5 adverse events.
